# Geographical alignment of prehospital critical care services with population need for out-of-hospital cardiac arrest patients

**DOI:** 10.1186/s13049-026-01655-9

**Published:** 2026-07-15

**Authors:** Adam J. Boulton, Nigel Lang, Joshua Miller, Gregory Adam Whitley, Oliver Zorab, Adam de Paeztron, Caroline Leech, Paramjit Gill, Gavin D. Perkins, João Porto de Albuquerque, Terry P. Brown, Joyce Yeung

**Affiliations:** 1 Warwick Clinical Trials Unit, Warwick Medical School, University of Warwick, Coventry, CV4 7AL UK; 2 Devon Air Ambulance, Exeter, UK; 3 West Midlands Ambulance Service University NHS Foundation Trust, Brierley Hill, UK; 4 Clinical Audit and Research Unit, East Midlands Ambulance Service NHS Trust, Lincoln, UK; 5 South Western Ambulance Service NHS Foundation Trust, Exeter, UK; 6 Dorset and Somerset Air Ambulance, Wellington, UK; 7 Out-of-Hospital Cardiac Arrest Outcome (OHCAO) Project, Clinical Trials Unit, Warwick Medical School, University of Warwick, Coventry, UK; 8 Emergency Department, University Hospitals Coventry and Warwickshire NHS Trust, Clifford Bridge Road, Coventry, UK; 9 The Air Ambulance Service, Blue Skies House, Butlers Leap, Rugby, UK; 10 Warwick Applied Health, Medical School, University of Warwick, Coventry, UK; 11 Critical Care Unit, University Hospitals Birmingham NHS Foundation Trust, Birmingham, UK; 12 Urban Big Data Centre, School of Social and Political Sciences, University of Glasgow, Glasgow, G12 8QQ Scotland, UK

**Keywords:** Emergency medical services, Resuscitation, Resource allocation, Socioeconomic factors, Geographic information systems, Health inequalities, Health inequities, Air ambulance, Prehospital physician, Advanced life support

## Abstract

**Background:**

Prehospital critical care is associated with improved outcomes following out-of-hospital cardiac arrest (OHCA), but may not be distributed equitably.

**Aim:**

To examine the alignment between prehospital critical care provision and neighbourhood OHCA risk, predicted survival, and deprivation across three neighbouring ambulance regions in England.

**Methods:**

Data on all OHCAs treated by three neighbouring English ambulance services (2018–2023) were aggregated to the neighbourhood level. Prehospital critical care response time predictions were generated from service-supplied operational parameters and calibrated against observed data with rural-urban adjustment. High-risk neighbourhoods were defined by above-median OHCA incidence and below-median bystander CPR. Pre-EMS predicted survival was estimated using a published model. Deprivation was measured with Index of Multiple Deprivation. Weighted regression models with service-by-response time interaction terms examined each neighbourhood metric per 5-minute response time increase. A secondary analysis examined a HEMS-only configuration by removing the unique urban, NHS car asset in service B.

**Results:**

Overall, 52.9% of the resident population and 51.3% of OHCAs were within 15 min of a prehospital critical care response. In two services, increasing response time was associated with higher odds of a neighbourhood being high-risk and with greater deprivation. For pre-EMS predicted survival, poor alignment was found in one service, favourable alignment in another, and no significant association in the third. Service B showed favourable alignment across all three metrics. Removing the urban car asset (HEMS-only configuration) reversed this favourable pattern, producing gradients consistent with the other two services. Sensitivity analysis using observed response times and distances produced consistent but attenuated associations.

**Conclusion:**

Prehospital critical care provision, particularly HEMS, is generally poorly aligned with neighbourhood OHCA risk, pre-EMS predicted survival, and deprivation. The only region demonstrating favourable cardiac arrest alignment was attributable to its urban NHS car asset. Expanding car-based prehospital critical care in deprived urban areas may warrant consideration as a strategy to reach more OHCA patients and improve equity of access.

**Supplementary Information:**

The online version contains supplementary material available at https://doi.org/10.1186/s13049-026-01655-9.

## Introduction

Prehospital critical care (PHCC) teams are routinely involved in the management of out-of-hospital cardiac arrest (OHCA) patients in modern advanced emergency medical services (EMS) [1]. These teams comprise experienced and expert clinicians who deliver an enhanced level of care beyond standard advanced life support (ALS). A recent International Liaison Committee on Resuscitation (ILCOR) review found that attendance of PHCC to OHCA patients was associated with improved outcomes, although the supporting evidence relies on non-randomised studies and the certainty of evidence was low [2]. The EMS organisation of PHCC and their attendance to OHCA is now included in European guidelines [3, 4]. 

Despite this, there are inequalities in access to PHCC following OHCA. A recent cohort study in England found that PHCC teams were attending over a quarter of all OHCA patients, however attendance rates varied across the three included ambulance regions (13.0% to 39.8%) [5]. Helicopter Emergency Medical Services (HEMS) provided by air ambulance charities accounted for 85.5% of PHCC attendances, responding by air in just over half. An urban, physician-led NHS team responding by car accounted for 5.1% of attendances. This study revealed that OHCA patients in more deprived neighbourhoods were less likely to be attended by PHCC. Subsequent spatial analysis demonstrated that this inequality was explained by the geographical configuration of services [6]. This is in keeping with evidence of regional and temporal inequalities in the provision of PHCC within the UK [7, 8]. 

These two studies demonstrate a deprivation-related inequality in PHCC attendance that is driven by how services are geographically configured. Both, however, described observed attendance relative to deprivation, addressing equality of provision. Efficient and equitable healthcare services are aligned to the disease risk within a population and therefore can provide care at an intensity to match the population need [9, 10]. Given the inequalities in more proximal links in the chain of survival [11–19], such alignment of PHCC could mitigate rather than reinforce inequalities [20, 21]. Whether PHCC is configured equitably and aligned with the underlying disease burden remains unknown. Addressing this requires moving beyond observed attendance to model response times across all neighbourhoods, including those with no PHCC attendance, and assessing these against OHCA risk and predicted survival alongside deprivation.

This study aimed to examine the alignment between prehospital critical care provision and neighbourhood cardiac arrest risk, predicted survival, and deprivation across three neighbouring ambulance regions in England.

## Methods

An observational spatial ecological design was used and the reporting follows the STrengthening the Reporting of OBservational studies in Epidemiology statement [22]. This study is part of a wider project with a pre-specified protocol, which received Health Research Authority and Research Ethics Committee approval (23/NW/0378), and was prospectively registered (researchregistry10307) [23]. 

The study uses the same data as previously published [5]. Briefly, three bordering NHS ambulance services in England were included, covering a geographical area of approximately 21,500 square miles with an estimated total population of 16.5 million. Service A (HEMS-only) borders service B (HEMS, plus urban, physician-led NHS car asset), and service B borders service C (HEMS-only). All services operate a tiered emergency response, with calls for suspected cardiac arrests receiving the highest priority (category 1) and assigned an immediate ALS-trained paramedic response. Additionally, PHCC teams may attend as dispatched by control room staff. PHCC dispatch may occur as: closest asset to a category 1 call; by immediate dispatch criteria (e.g. major incident, traumatic cardiac arrest, maternal cardiac arrest); by interrogated dispatch criteria (after obtaining additional information from the caller); or as a direct request from on-scene ambulance crew. PHCC was defined as any provider with clinical competencies beyond that of standard paramedics following Joint Royal Colleges Ambulance Liaison Committee (JRCALC) guidelines. The PHCC resources within each participating ambulance region are summarised in the Supplementary Material.

All patients sustaining an OHCA with attempted resuscitation by an NHS ambulance service between 1st January 2018 and 31st December 2023 were included. This included all ages and all aetiologies of cardiac arrest (including traumatic). Individual patient-level data was obtained from the Out-Of-Hospital Cardiac Arrest Outcomes (OHCAO) registry, NHS ambulance services, and one independent air ambulance charity. Clinical variables and location of event were obtained from the OHCAO registry as previously described [5, 6, 24]. Witnessed status and bystander CPR were combined into one variable to account for EMS witnessed cases being considered as not receiving bystander CPR within the OHCAO registry and leading to potentially misleading results [25]. Data concerning PHCC response time was obtained from the three participating NHS ambulance services, and supplemented with data from one independent air ambulance charity. Expert research clinicians at each site identified PHCC assets responding within their ambulance service region, including HEMS resources, NHS ambulance service resources, or British Association for Immediate Care Schemes (BASICS) provided by volunteer professional critical care responders. The Lower Layer Super Output Area (LSOA) was obtained for the location of event, with corresponding data for Index of Multiple Deprivation (IMD) retrieved from the UK government [26]. IMD deciles were used unprocessed, with lower deciles indicating higher levels of deprivation. IMD was released in 2019 and mapped to LSOA11 codes. As the UK Census was updated in 2021, new LSOA21 codes were generated for some neighbourhoods. In the study dataset, the IMD was not immediately available for 17 LSOAs. A lookup table from the Office for National Statistics (ONS) was used to obtain the exact fit LSOA11 codes for these LSOA21 neighbourhoods with missing IMD. This allowed IMD to be obtained for all neighbourhoods in the dataset. The 2021 rural-urban classification at the LSOA level was obtained from the ONS [27]. 

Pre-EMS patient variables were used to estimate individual patient survival to hospital discharge using a previously validated risk adjustment model [28]. This published model performed well in development and validation cohorts (area under the receiver operating characteristic curve 0.877 and 0.871, respectively). The variables used were patient’s age, aetiology, witnessed/bystander CPR status, and initial rhythm. Individual predicted survival probabilities were aggregated to the neighbourhood level using the median, as individual values were heavily right-skewed (mean 8.5%, SD 13.1%; median 1.9%, IQR 0.9% to 9.6%), such that 75% of patients had a pre-EMS predicted survival below 10%, making the median a more representative measure of the local case-mix than the mean.

The study region was mapped using spatial boundary files and all neighbourhoods (LSOAs) within this region were identified. Individual patient data was aggregated to the neighbourhood-level, generating neighbourhood counts of total number of OHCAs and continuous variables of median observed PHCC response time, proportion of cases receiving bystander CPR (denominator was bystander-witnessed cases), and the median pre-EMS predicted survival probability. From these aggregated measures, high-risk neighbourhoods were defined as those with above-median OHCA incidence and below-median bystander CPR rate (study region medians). This approach is consistent with previous national analyses to identify high-risk neighbourhoods and inform targeted public health initiatives [14, 29]. Annual OHCA incidence per 100 000 population was calculated using 2021 UK Census resident population from NOMIS web [30]. 

Individualised predicted PHCC response times were generated for all neighbourhoods. This included helicopter and road responses to reflect the mixed response model used in the UK. Response time was defined as the interval from PHCC activation to arrival on scene, to ensure comparability between response modalities, given the differences in activation‑to‑mobilisation intervals between modalities. All PHCC services in the study region supplied base-specific operational parameters (helicopter model, lift time, cruise speed), alongside typical response mode (road or helicopter) to areas near to HEMS bases to reflect base-specific operational norms given local geography and receiving hospitals. Helicopter times were estimated from Euclidean distance and calibrated against observed response times using linear regression with rural-urban adjustment. This calibration step accounted for landing and patient access time in an adjusted and data-driven manner. Road times were generated using Open Source Routing Machine and calibrated against observed activation-to-scene times to give emergency response driving times. Full details of the prediction methodology are provided in the Supplementary Material. The prediction model performed well, with a mean absolute error of 3.49 min and root mean squared error of 4.67 min.

Descriptive statistics were produced for the individual patients entering the study, using counts with percentages for categorical variables and means with standard deviations (SD) or medians with inter-quartile ranges (IQR) for continuous variables. Regression models were constructed to examine the relationship between predicted response times and each neighbourhood metric per 5-minute increase in response time to aid interpretability. Models with a service–response time interaction were used as the primary analysis as they reflected the differing PHCC provisions, enabled estimation of service‑specific odds ratios, and consistently produced the best fit based on AIC compared with mixed‑effects and non‑interaction models (Supplementary Material). Models using high-risk neighbourhood and pre-EMS predicted survival were weighted by neighbourhood OHCA count to ensure areas contributed in proportion to their case volume. Rural-urban classification was included as a covariate due to differing PHCC availability and response, which also improved AIC (Supplementary Material). Models without rural-urban adjustment were conducted as sensitivity analyses. Models with adjustment for EMS response time were also conducted as sensitivity analyses. IMD was modelled as an ordinal variable in the primary analysis, reflecting its construction as a ranked deprivation measure. Continuous IMD was included as a sensitivity analysis. The primary analysis used individualised PHCC predicted response times. The secondary analysis examined a HEMS-only configuration by removing the urban NHS car asset from service B. Sensitivity analyses used the subset of neighbourhoods where observed PHCC response times were available, road distance to nearest PHCC base, and straight-line distance to nearest HEMS base. An additional sensitivity analysis was conducted excluding cases occurring during the COVID-19 pandemic peaks. Linearity of the logit was assessed, and collinearity was examined using variance inflation factors. As a model diagnostic, residual spatial autocorrelation was examined using Moran’s I statistic applied to model residuals, with spatial weights derived from queen contiguity neighbourhood relationships. (Supplementary Material). The magnitude of Moran’s I was negligible for high-risk neighbourhood and pre-EMS predicted survival models (0.01 to 0.03), but much higher for deprivation models (0.5), reflecting the well-known geographic clustering of IMD in England [31, 32]. A spatial error model was therefore fitted as a sensitivity analysis for deprivation only. All data processing, analysis, and visualisation was performed using R with the following packages active: broom, car, emmeans, leaflet, lme4, MASS, mice, spatialreg, spdep, sf, tidyverse, tmap [33]. 

## Results

The study region encompassed 10,032 neighbourhoods, of which 9,639 recorded at least one OHCA event between 1st January 2018 and 31st December 2023. This encompassed 57,601 OHCA patients and the descriptive characteristics of these patients are reported in Supplementary Material.

**Fig. 1:**
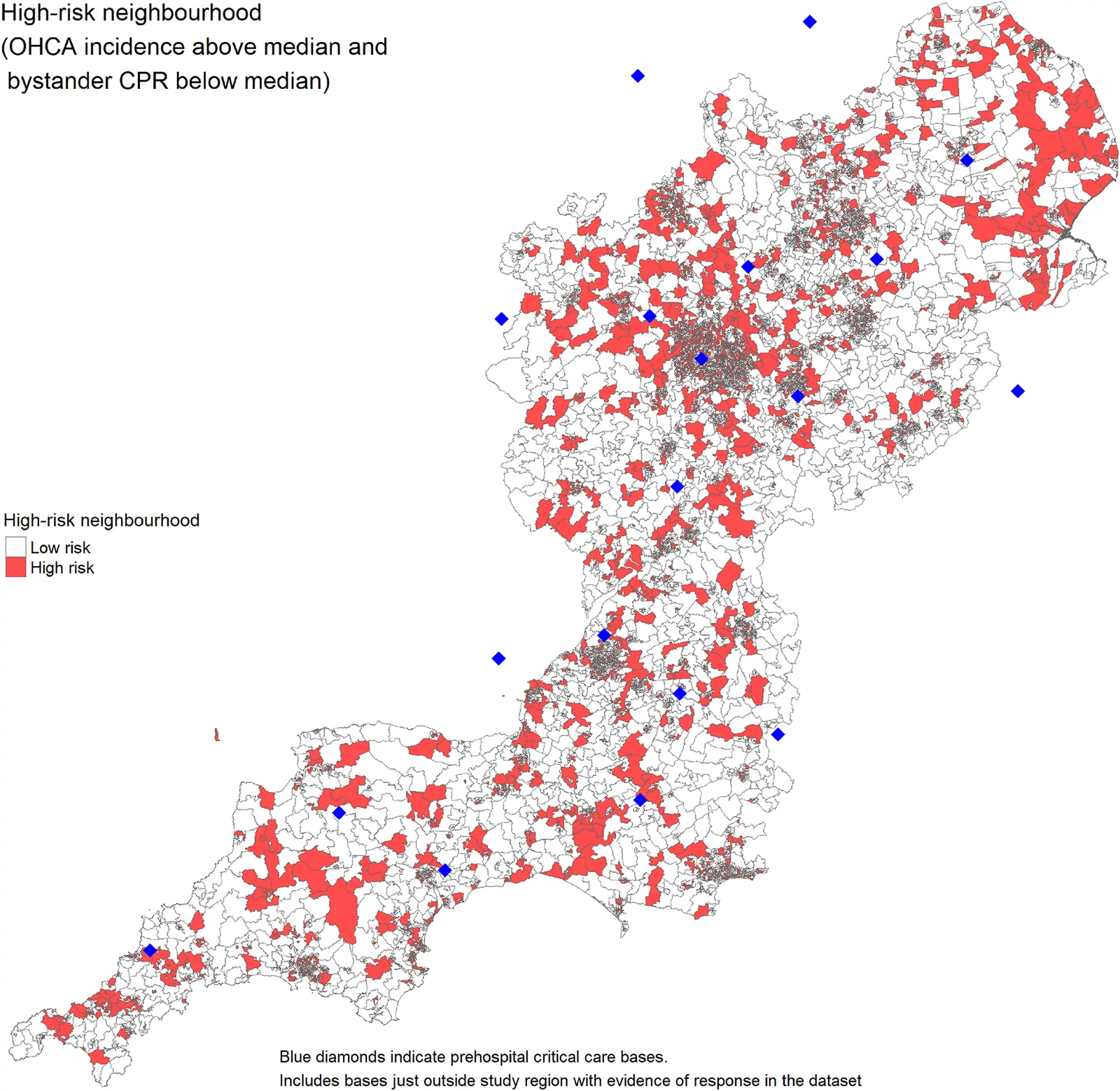
Map of high-risk neighbourhoods (out-of-hospital cardiac arrest incidence above the median and bystander CPR below the median). Presented at the Lower Layer Super Output Area (LSOA) level. Prehospital critical care bases indicated by blue diamonds and includes bases outside of the ambulance region with evidence of attendance to cardiac arrest cases in the dataset. An interactive version is available online (Interactive high-risk map ; https://high-risk.netlify.app/)

**Fig. 2:**
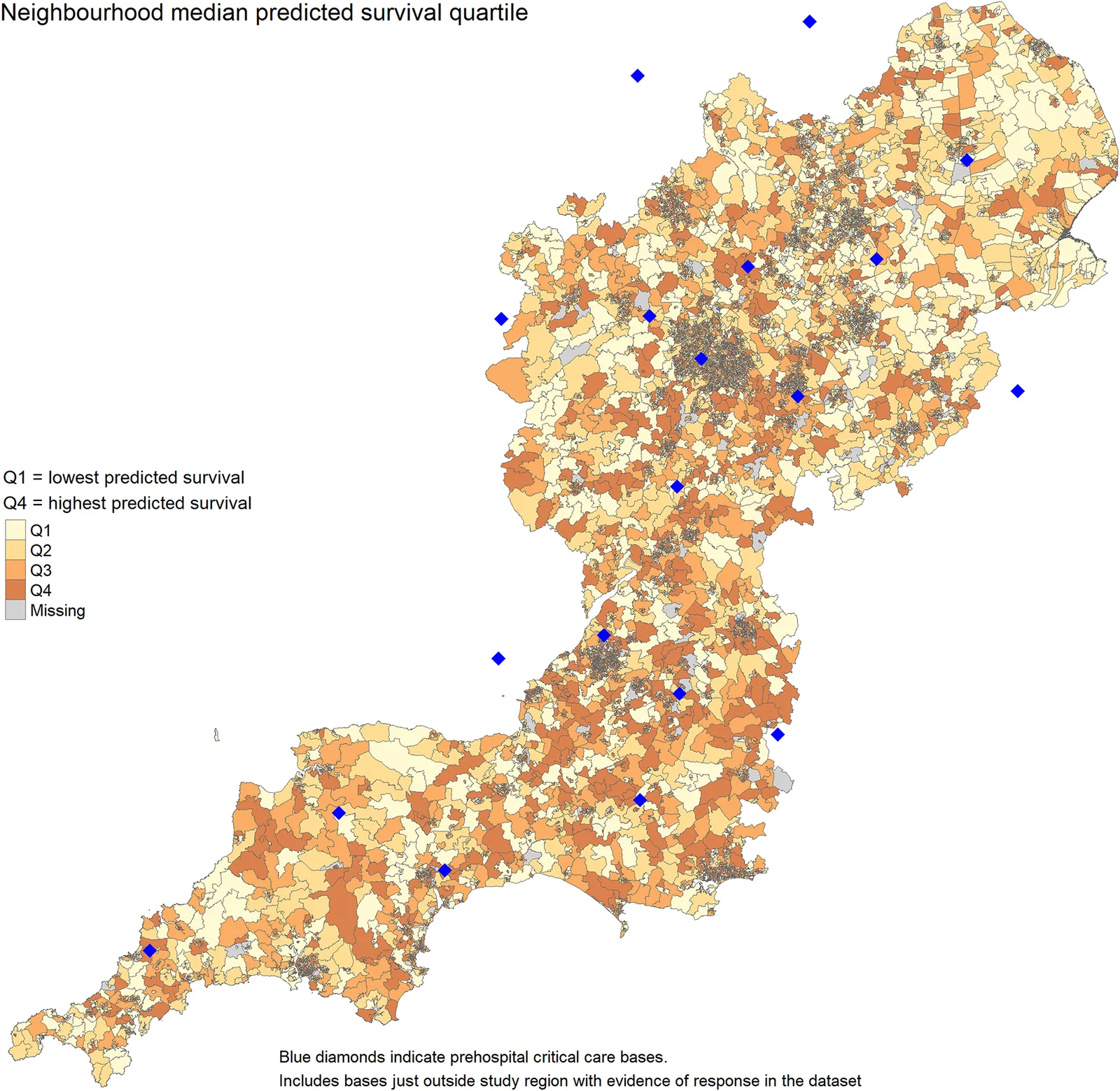
Map of neighbourhood pre-EMS predicted survival. Individual patient predicted survival was produced with a validated model using pre-EMS intervention variables, and then aggregated at the Lower Layer Super Output Area (LSOA) level. An interactive version is available online (https://predicted-survival.netlify.app/)

The median number of OHCAs in each neighbourhood was 5 (IQR 3 to 8), with a median incidence of 54.82 per 100,000 resident population (IQR 36.25 to 77.11). A total of 2,824 (28.1%) neighbourhoods were identified as high-risk, defined as an OHCA incidence above the median and a proportion bystander CPR below the median. The neighbourhood median pre-EMS predicted survival was 2.1% (IQR 1.3% to 4.4%). These are mapped in Figs. 1 and 2.

**Fig. 3:**
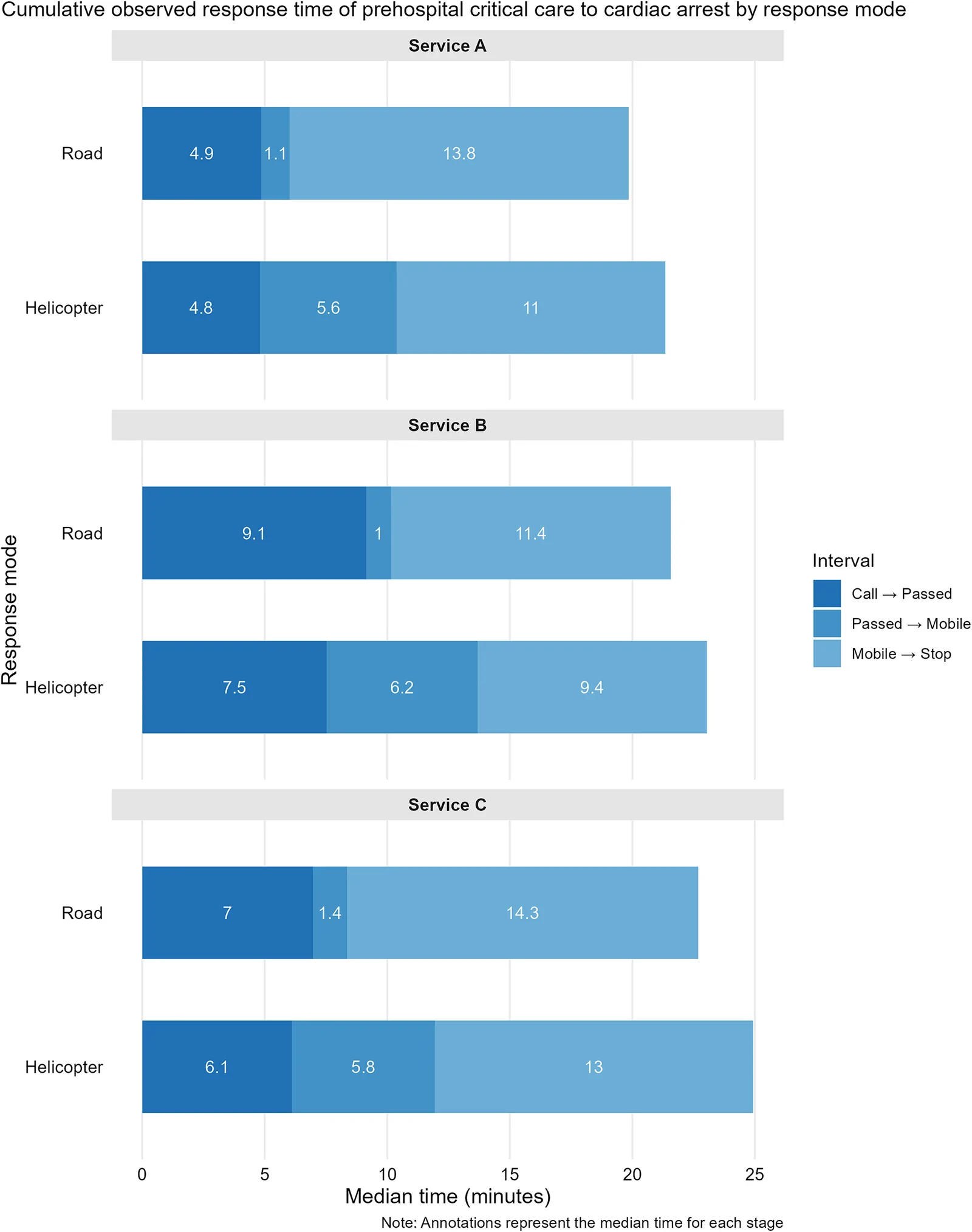
Cumulative observed response time of prehospital critical to cardiac arrest across services

A PHCC asset attended an OHCA in 6,731 neighbourhoods. Observed response time data was available for 17,244 PHCC responses across all 6,731 neighbourhoods. The description of PHCC response and distribution of response times is presented in the Supplementary Material. For helicopter response, the median time from EMS call connected to ambulance service to tasking of PHCC was 6.3 min (IQR 3.7 to 15.0), from tasking to PHCC being mobile was 5.9 min (IQR 4.2 to 7.6), and from mobile to stopping at scene was 12.0 min (IQR 8.1 to 15.9). For road response, the median time from EMS call to tasking of PHCC was 7.4 min (IQR 3.8 to 17.8), from tasking to PHCC being mobile was 1.2 min (IQR 0.2 to 2.4), and from mobile to stopping at scene was 12.8 min (IQR 8.4 to 18.6). These call intervals stratified by service are visualised in Fig. 3.

**Fig. 4:**
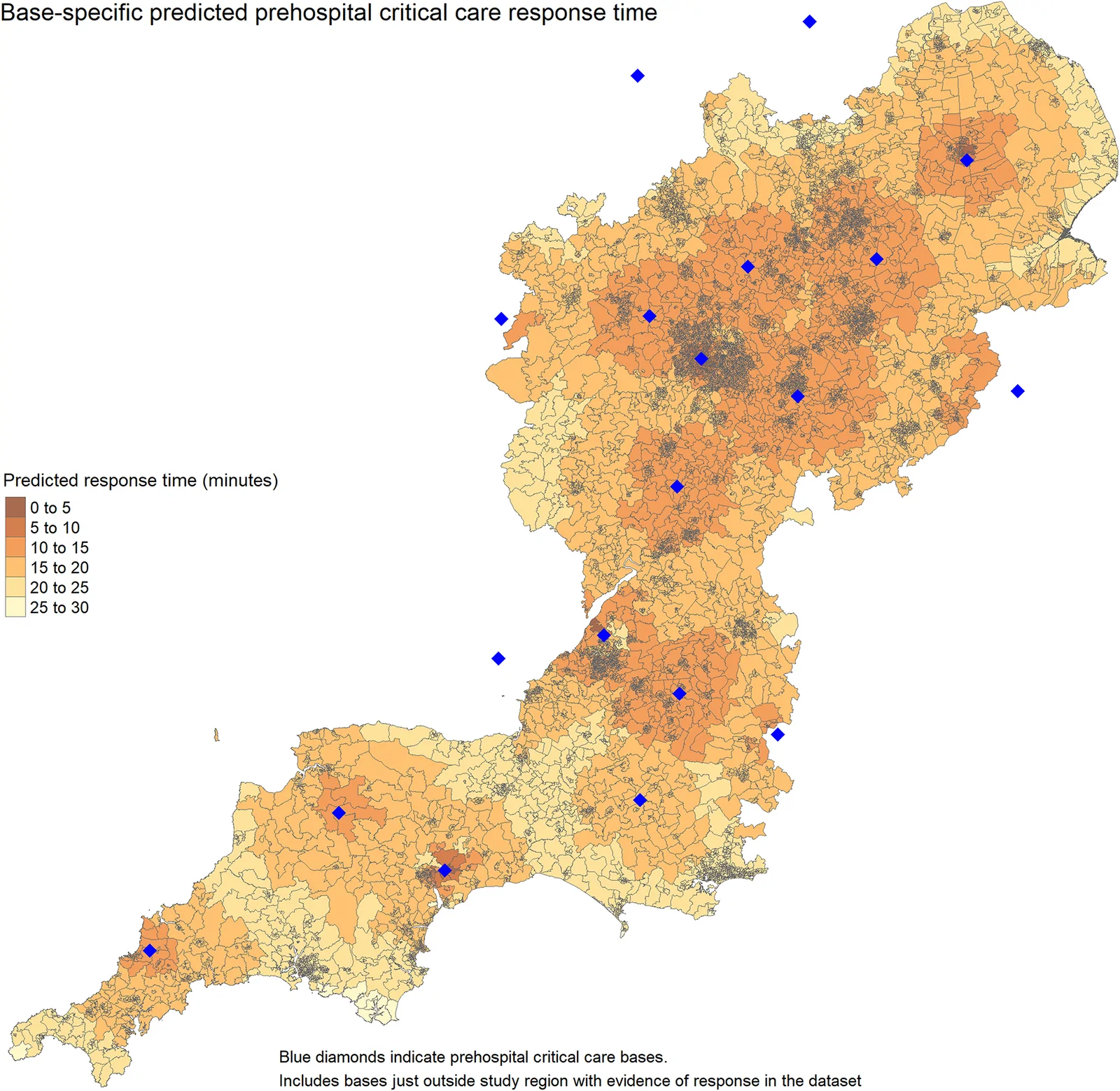
Map of base-specific predicted prehospital critical care response times

**Fig. 5:**
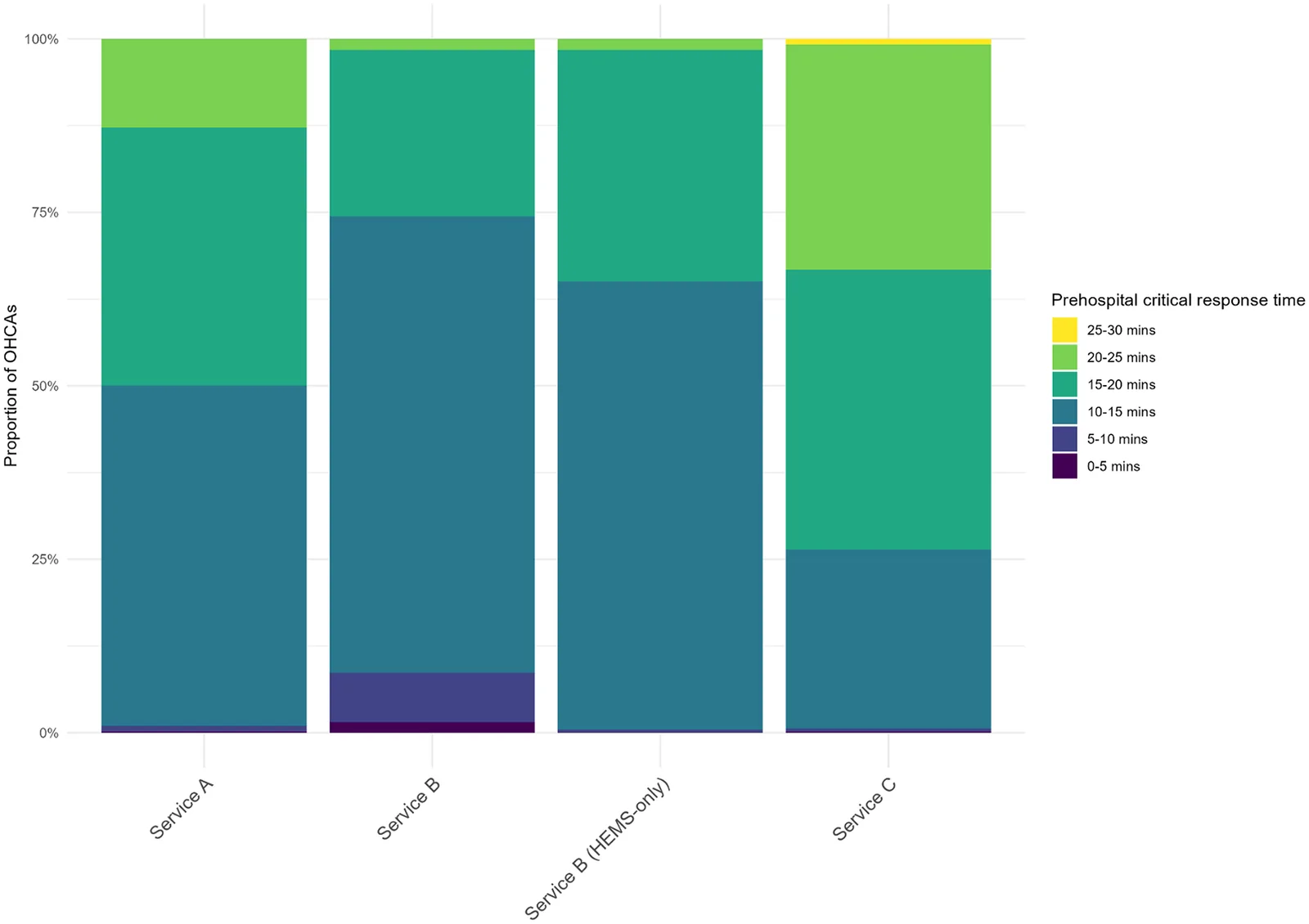
Proportion of total cardiac arrests by prehospital critical care response time. HEMS: Helicopter emergency medical service; OHCA: Out-of-hospital cardiac arrest; PHCC: Prehospital critical care. Service B includes two columns: the standard configuration including all PHCC assets, and a HEMS‑only configuration in which the unique land-based NHS asset has been removed. Services A and C operate only HEMS-based PHCC assets and therefore represent HEMS-only response times

The predicted response time model performed well and demonstrated good calibration against observed response times (Supplementary Material). The neighbourhood response times are mapped in Fig. 4 and the distribution visualised in the Supplementary Material. The median was 14.75 min (IQR 13.01 to 17.51). Overall, the resident population residing within 15 min of a PHCC response was 8,914,730 (52.9%). The population distribution across response time bands varied between services (Supplementary Material). In service B, removal of the urban NHS car asset reduced the resident population within 15 min from 4,480,736 (74.6% of service B resident population) to 3,926,322 (65.4%). Across the study period, 29,566 OHCAs (51.3%) occurred in a neighbourhood within 15 min of a PHCC response. In service B, this decreased from 17,203 (74.4% of OHCAs in service B) to 15,032 (65.0%) when the urban NHS car asset was removed. The distribution of OHCAs across PHCC response times varied (Fig. 5).

**Fig. 6:**
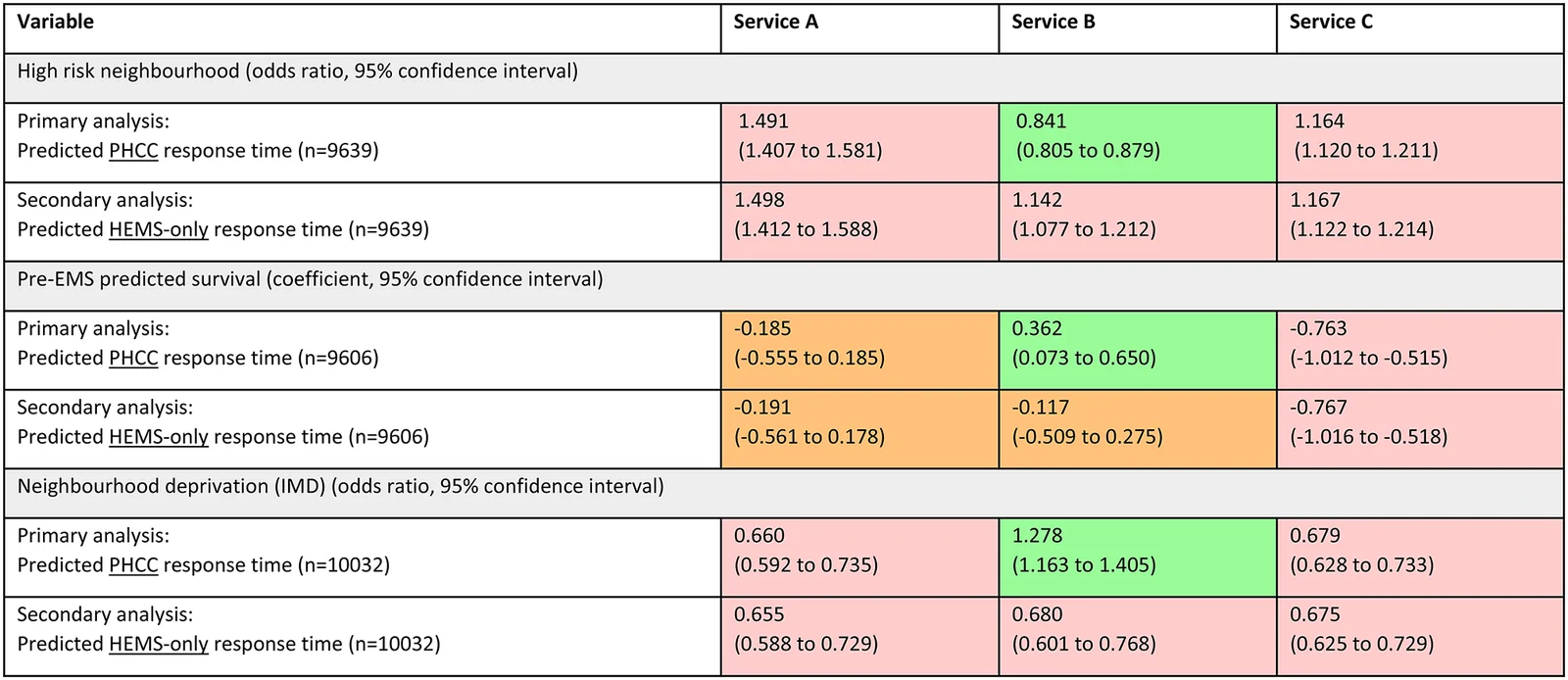
Alignment of prehospital critical care response time with neighbourhood characteristics. Green = favourable alignment; Amber = no significant relationship; Red = poor alignment. Individualised predicted PHCC response times are assigned from each neighbourhood’s nearest PHCC base across three ambulance service regions, with response mode (road or helicopter) determined by base-specific operational norms reflecting local practice and geography. Predictions incorporate base-level helicopter parameters (lift time, cruise speed) and are calibrated against observed times using linear regression with a rural-urban adjustment. The HEMS-only configuration removes the urban NHS car asset from Service B. Effect estimates are per 5-minute increase in response time. IMD: Index of multiple deprivation. For high‑risk neighbourhood, an OR >1 indicates higher chance of being a high‑risk neighbourhood per 5‑minute increase in response time. For neighbourhood pre-EMS predicted survival, coefficients represent the percentage‑point change in predicted survival per 5‑minute increase in response time. For IMD decile (1=most deprived, 10=least deprived), an OR >1 indicates the neighbourhood being less deprived per 5-minute increase in response time; OR <1 indicates the neighbourhood being more deprived per 5-minute increase in response time

Associations between response time and all neighbourhood characteristics differed significantly by service (interaction *p* < 0.001). The results of the primary and secondary analysis across all neighbourhood characteristics are displayed in Fig. 6, coloured to illustrate favourable or unfavourable alignment.

### High-risk neighbourhood

In the primary analysis of individualised PHCC predicted response time, services A and C showed poor alignment between response time and high-risk neighbourhood status, with longer response times associated with increased odds of a neighbourhood being classified as high-risk. Service B showed the opposite, with shorter response times associated with higher odds of high-risk, indicating favourable alignment. The secondary analysis of a HEMS-only configuration reversed this favourable pattern in service B, producing unfavourable alignment consistent with services A and C.

### Pre-EMS predicted survival

For pre-EMS predicted survival, service C showed poor alignment in the primary analysis, with longer response times associated with poorer predicted survival. Service B showed favourable alignment, with shorter response times associated with worse predicted survival. Service A showed no significant association with pre-EMS predicted survival. In the secondary analysis of a HEMS-only configuration, the favourable association in service B was lost, with all three services showing no significant or unfavourable alignment.

### Neighbourhood deprivation

For deprivation, services A and C showed poor alignment in the primary analysis, with longer response times directed toward more deprived neighbourhoods. Service B showed favourable alignment. As with the other metrics, the secondary analysis of HEMS-only configuration reversed this in service B, with all three services then showing consistent poor alignment.

### Sensitivity analysis

Sensitivity analysis using observed response times, road distance to nearest PHCC base, and straight-line distance to nearest HEMS bases produced directionally consistent but attenuated findings throughout (Supplementary Material). A sensitivity analysis excluding cases from COVID-19 pandemic peaks produced almost identical results. Sensitivity analysis with adjustment for EMS response time also produced almost identical results. Model structure sensitivity analyses of no rural-urban adjustment, treating IMD as a continuous variable, and a spatial error model for IMD also produced consistent results.

## Discussion

Across three ambulance services, the geographical configuration of PHCC was generally not well aligned with neighbourhood OHCA risk, pre-EMS predicted survival, or socioeconomic deprivation. Only one service demonstrated a provision in which shorter PHCC response times were directed toward neighbourhoods with greater need. However, this pattern appeared driven by the presence of an urban NHS car asset. Once this team was removed and the HEMS‑only configuration investigated, the service demonstrated the same inverse or neutral gradients observed elsewhere. Sensitivity analysis using distances and the subset of neighbourhoods with observed response times produced consistent, albeit attenuated, results, reinforcing the reliability of the modelling approach. The use of base-specific predicted PHCC response times improves the reliability and generalisability of these findings, as well as offering a potential avenue to optimise services and measure the likely effect of alternative configurations.

The suite of advanced interventions provided by PHCC for OHCA patients is expanding and offers promise [34–39]. PHCC attendance is already associated with improved outcomes and is recommended in recent guidelines [1, 3, 4]. However, since OHCA outcomes are highly time-sensitive, PHCC services must be well positioned to reach patients rapidly in order to realise the full potential of these interventions [40–43]. Further, PHCC is a scarce and costly resource. Aligning the system to the disease risk offers a potential avenue to reach more OHCA patients in a timely fashion and use limited resources efficiently. This approach is consistent with the principle of proportionate universalism, a mechanism to tackle health inequalities by delivering services universally and at a scale and intensity proportionate to the population need [20, 44, 45]. A system in which PHCC response times are shortest in neighbourhoods with the highest OHCA incidence, lowest bystander CPR, poorest predicted survival, and greatest socioeconomic deprivation would represent a service well configured to disease risk. In this study, such favourable alignment was found in only one ambulance region, driven by the presence of an NHS-delivered car PHCC asset located within a deprived urban environment. When this asset was excluded and only HEMS resources were considered, the pattern resembled that of the other regions, with shorter PHCC response times in less deprived and lower risk neighbourhoods. PHCC responds to a broad spectrum of time-critical emergencies in addition to OHCA that are not considered in this analysis. Socioeconomic deprivation is also independently associated with high-energy and penetrating trauma [46, 47]. OHCA serves as a robust, well-characterised tracer condition for evaluating geographical alignment, given its acute time-sensitivity and the availability of high-quality registry data [48]. As deprivation drives need across OHCA, trauma, and other critical illness, a configuration aligned to OHCA risk is likely to benefit the wider time-critical PHCC workload rather than serving a single condition.

This study demonstrates the opportunity for land-based PHCC assets to improve access. Previous studies modelling optimisation of PHCC coverage has largely focused on helicopter assets, reflecting the large rural geographies of the regions previously investigated [49–55]. In the UK, an optimal configuration will utilise the complementary strengths of both helicopter and car assets given the current mixed response model. Helicopter assets can cover large areas and bridge long distances rapidly, but are costly and may not be available in adverse weather or at night [5, 7]. Their base locations are relatively fixed and influenced by numerous constraints, including aviation-related considerations, which explains the historical predominance of airports and airfields as base locations [56]. In contrast, there is greater flexibility in the location of car assets, which could operate from strategically placed mobile or satellite standby points, including existing fire stations or ambulance hubs. This study also highlights the potential for inequalities when PHCC provision relies on helicopters and the charitable structures that underpin them [57]. The favourable alignment observed in service B was driven by its publicly funded, urban NHS car PHCC team. This asset accounted for a small proportion of overall PHCC attendances (5.1%), reflecting its single-vehicle, urban remit. A single, strategically located car asset was sufficient to reverse the alignment gradient seen elsewhere, demonstrating the disproportionate equity impact achievable from modest, well-placed land-based capacity. However, whether this single asset can reach all eligible patients remains unexplored. Expansion of similar services in other regions may allow more OHCA patients to be reached by PHCC and address inequalities. The response time prediction methods developed in this study provide a practical, data-driven approach to identifying optimal satellite locations for these car assets, enabling services to model multiple candidate configurations and assess their likely impact on coverage and equity before implementation. Broader data sharing and real-time operational refinement could support continual adaptation as cross-service provision, dispatch patterns, and OHCA case-mix evolve, and populations change. This data-informed integration of strategically placed car assets within the existing PHCC model could create a more agile system that maximises efficiency, delivers scarce PHCC resources proportionately to population need, and allows services to reach more patients rapidly and ultimately save more lives.

Further work should seek to validate these findings across other systems and configurations since the present analysis reflects a specific regional and temporal context. Prospective validation of the response-time prediction model would allow further refinement of the model and support its use for planning. Where reconfiguration occurs, continual assessment of patient-level outcomes would clarify how geographical alignment translates into clinical benefit, rather than relying on predicted survival and risk surrogates. Finally, the predicted response times reflect the geographical configuration of assets and do not incorporate time taken for dispatch decision-making. This study found the interval between EMS call and PHCC tasking to be a substantial and variable component of the total response time. The impact of approaches to dispatch on time to tasking and subsequent alignment therefore demands further exploration [58]. 

### Strengths and limitations

A strength of this study is the development of individualised, base-specific PHCC response-time predictions that were calibrated using real response-time data and validated against a subset of observed times. Previous optimisation studies have typically modelled helicopter assets alone and applied uniform assumptions across bases without calibration or assessment against observed response times [49, 59, 60]. The individualised approach used here reflects the mixed UK response model and incorporates base-level variation in helicopter lift times, cruise speeds, and local operational practices influencing whether a road or helicopter response is used to nearby neighbourhoods. Together, these features result in estimates that more closely represent operational practice. Adjustment for rural-urban status further improves reliability by accounting for differences in travel speeds, road networks, and scene access. The use of pre-EMS predicted survival from a validated model [28], alongside neighbourhood OHCA risk and deprivation, provides a broader assessment of pre-EMS patient risk and population need, strengthening the equity focus of the analysis. Its small effect size is a reflection of the low absolute survival probabilities when aggregated at a neighbourhood-level. The robustness of findings is supported by a range of sensitivity analyses examining alternative exposure variables of actual response times and distance, as well as alternative model structures, which produced directionally consistent results throughout.

This study has several limitations. The findings reflect the PHCC provision and configuration within the three ambulance service regions during the study period and may not generalise to other systems or future configurations. The favourable alignment in one region was driven by a single urban car asset, so this observation, while consistent across all three metrics, derives from one service and should be interpreted as hypothesis-generating rather than confirmatory. This study models geographical alignment rather than patient outcomes, so whether improved alignment translates into survival benefit remains to be demonstrated. This study examines alignment in terms of OHCA, using it as a tracer condition, however PHCC respond to a broad spectrum of other time-critical emergencies. Predicted response times assume unrestricted helicopter flying conditions and that the nearest asset responds, whereas real‑world availability varies with weather, tasking conflicts, and night-time operating restrictions. Therefore, this represents a best‑case scenario and may underestimate the disparities in access, particularly at night when overall PHCC assets and helicopter availability are reduced [7]. The modelling does not account for assets starting away from base, such as during follow-on taskings, and some bases may have multiple co‑located assets that cannot be disentangled in this analysis. Neighbourhood centroids and aggregated data were used, which is necessary for travel time predictions and population‑level analysis but may introduce spatial misclassification [61]. Sensitivity analyses using observed response times were limited to neighbourhoods where PHCC attended an OHCA, introducing bias. Missingness in observed response time is greatest in regions with lower attendance rates, reducing the accuracy of these estimates. BASICS assets were not included in the model since their availability and location varied across the study period, in addition to their responding locations including volunteer private home addresses. HEMS using car assets on mobile or temporary satellite standby points could not be incorporated because their availability and locations varied over the study period. Finally, analyses were conducted at neighbourhood level and therefore associations may not directly represent individual-level relationships, a limitation inherent to ecological study designs.

## Conclusions

PHCC services in the studied region were generally poorly aligned with neighbourhood OHCA risk, pre-EMS predicted survival, and socioeconomic deprivation. The only configuration demonstrating alignment with cardiac arrest population need was attributable to an urban NHS car asset. When removed, this region exhibited the same unfavourable gradients as the others. These findings support consideration of strategic optimisation of PHCC configuration, with particular attention to urban car capacity, satellite standby points, and alignment with neighbourhood risk to facilitate efficient use of scarce resources. As PHCC teams respond to a broad spectrum of time-critical emergencies, optimisation should ultimately reflect this wider case-mix rather than cardiac arrest alone, though socioeconomic deprivation is a shared driver across these conditions and alignment to OHCA risk is therefore likely to benefit the wider workload. The individualised response‑time modelling developed here provides a potential tool for future optimisation, enabling services to maximise coverage efficiently, address inequalities, and improve the ability to reach critically ill patients rapidly.

## Supplementary Information

Below is the link to the electronic supplementary material.

Supplementary Material 1

## Data Availability

The dataset used for this study was obtained from the Out-of-Hospital Cardiac Arrest Outcomes (OHCAO) registry at the University of Warwick. Requests for access to data should be made to the OHCAO registry team (Contact: ohcao@warwick.ac.uk).

## Abbreviations

AIC: Akaike Information Criterion
BASICS: British Association for Immediate Care Schemes
CI: Confidence Interval
CPR: Cardiopulmonary Resuscitation
EMS: Emergency Medical Service
HEMS: Helicopter Emergency Medical Service
ILCOR: International Liaison Committee on Resuscitation
IMD: Index of Multiple Deprivation
IQR: Interquartile Range
LSOA: Lower Layer Super Output Area
NHS: National Health Service
OHCA: Out-of-Hospital Cardiac Arrest
OHCAO registry: Out-of-Hospital Cardiac Arrest Outcomes registry
OR: Odds Ratio
PHCC: Prehospital Critical Care
SD: Standard Deviation
UK: United Kingdom
