# Supplementary material for: Geographical alignment of prehospital critical care services with population need for out-of-hospital cardiac arrest patients
Source: Scand J Trauma Resusc Emerg Med. 2026 Jul 15;34:164. doi: 10.1186/s13049-026-01655-9 (PMC13660141; doi:10.1186/s13049-026-01655-9)
Supplement: Supplementary file 1 [file 13049_2026_1655_MOESM1_ESM.docx]

Geographical alignment of prehospital critical care services for out-of-hospital cardiac arrest patients

Supplementary material

Contents

[Service prehospital critical care provision	2](#_Toc233532897)

[Study flow diagram	3](#_Toc233532898)

[Individual patients entering study	4](#_Toc233532899)

[Missing data description	5](#_Toc233532900)

[Individual cases with missing data	5](#_Toc233532901)

[Neighbourhood aggregated data missing data	6](#_Toc233532902)

[Neighbourhood-level patient characteristics stratified by quartile of median predicted survival	7](#_Toc233532903)

[Observed prehospital critical care response	8](#_Toc233532904)

[Description of observed response	8](#_Toc233532905)

[Response times	9](#_Toc233532906)

[Histogram of observed prehospital critical care response times (from activation to on scene time)	9](#_Toc233532907)

[Histogram of observed helicopter response times (from activation to on scene time)	10](#_Toc233532908)

[Histogram of observed road response times (from activation to on scene time)	11](#_Toc233532909)

[Map of observed prehospital critical care response times (from activation to on scene time)	12](#_Toc233532910)

[Quintile map of observed prehospital critical care response times (from activation to on scene time)	13](#_Toc233532911)

[Base-specific predicted prehospital critical care response times	14](#_Toc233532912)

[Predicted versus observed response times	15](#_Toc233532913)

[Histogram of predicted prehospital critical care response times	17](#_Toc233532914)

[Population distribution by prehospital critical care response time	18](#_Toc233532915)

[Population distribution by prehospital critical care response time across services	19](#_Toc233532916)

[Population distribution by HEMS-only configuration response time in service B	20](#_Toc233532917)

[Model performance across various model structures	21](#_Toc233532918)

[Model performance across analyses	22](#_Toc233532919)

[Model diagnostic: Spatial autocorrelation in raw outcomes and model residuals	23](#_Toc233532920)

[Sensitivity analyses	24](#_Toc233532921)

[Sensitivity analysis: observed response time and distances	24](#_Toc233532922)

[Sensitivity analysis: excluding cases from COVID-19 pandemic peaks	25](#_Toc233532923)

[Sensitivity analysis: with adjustment for EMS response time	26](#_Toc233532924)

[Sensitivity analysis: without adjustment for rural-urban classification	27](#_Toc233532925)

[Sensitivity analysis: Modelling of Index of Multiple Deprivation as a continuous variable	28](#_Toc233532926)

[Sensitivity analysis: Spatial error model of Index of Multiple Deprivation as a continuous variable	29](#_Toc233532927)

# Service prehospital critical care provision

| **Service** | **Summary of prehospital critical care provision** |
| --- | --- |
| Service A | Two Helicopter Emergency Medical Services within region   - Charity 1: One base within region with helicopter and road response capabilities - Charity 2: One base within region with helicopter and road response capabilities   Two BASICS schemes |
| Service B | Two Helicopter Emergency Medical Services within region   - Charity 1: Three bases within region with helicopter and road response capabilities - Charity 2: One base within region with helicopter and road response capabilities   One NHS ambulance service prehospital critical care team: One base with road response capability  Three BASICS schemes |
| Service C | Five Helicopter Emergency Medical Services within region   - Charity 1: Two bases within region with helicopter and road response capabilities - Charity 2: One base within region with helicopter and road response capabilities - Charity 3: One base within region with helicopter and road response capabilities - Charity 4: One base within region with helicopter and road response capabilities - Charity 5: One base within region with helicopter and road response capabilities   Eight BASICS schemes |

BASICS schemes confirmed from <https://www.basics.org.uk/schemes/> (accessed February 2026)

All Helicopter Emergency Medical Services were charity funded. The BASICS schemes were charity funded and delivered by volunteer professional critical care responders. The NHS ambulance service prehospital critical care asset in service B was NHS funded.

# Study flow diagram

OHCA patients in included ambulance service regions (Jan 2018-Dec 2023) within OHCAO registry

n = 61,011

Location of event confirmed to be within ambulance service region using geospatial filtering

n = 57,601

Location of event not missing

n = 57,675

Data aggregated to each LSOA

LSOA N = 9,639 with an OHCA event.

(LSOA in region 10,032)

# Individual patients entering study

Description of included out-of-hospital cardiac arrest patients. CPR: Cardiopulmonary resuscitation, EMS: Emergency medical services; IQR: Interquartile range; PEA: Pulseless electrical activity; SD: Standard deviation; VF: Ventricular fibrillation; VT: Ventricular tachycardia

|  | **All out-of-hospital cardiac arrests confirmed within the study region between 2018 and 2023 (N=57,601)** |
| --- | --- |
| Age – median (IQR)  Missing – N (%) | 70 (57–80)  282 (0.5%) |
| Sex – N (%)  Male  Female  Missing | 37,533 (65.2%) 19,968 (34.7%) 100 (0.2%) |
| Utstein location – N (%)  Place of residence  Education  Other  Public building  Sports  Street/Highway  Workplace  Missing | 47,193 (81.9%) 47 (0.1%)  6,098 (10.6%) 1,464 (2.6%)  167 (0.3%)  1,870 (3.3%) 440 (0.8%) 322 (0.6%) |
| Aetiology – N (%)  Medical  Asphyxia  Drowning  Overdose  Traumatic  Other (non-cardiac)  Missing | 53,088 (92.2%) 1,313 (2.3%) 233 (0.4%) 872 (1.5%) 1,477 (2.6%) 563 (1.0%) 55 (0.1%) |
| Initial rhythm – N (%)  Shockable (VF/VT)  Asystole  PEA  Missing | 12,897 (22.4%) 30,061 (52.2%) 11,540 (20.0%) 3,103 (5.4%) |
| Witness/bystander CPR – N (%)  Unwitnessed and no bystander CPR  Unwitnessed and bystander CPR provided  Bystander witnessed and bystander CPR provided  Bystander witnessed and no bystander CPR  EMS witnessed  Missing | 7,725 (13.4%) 13,945 (24.2%) 22,655 (39.3%) 6,911 (12.0%) 6,204 (10.8%) 161 (0.3%) |
| EMS call time – N (%)  0600-1200  1200-1800  1800-0000  0000-0600  Missing | 17,596 (30.5%)  16,383 (28.4%)  14,304 (24.8%)  8,346 (14.5%)  972 (1.7%) |
| Neighbourhood Index of Multiple Deprivation decile – mean (SD)  Missing | 5.1 (2.8)  0 (0.0%) |

There were 1,092 paediatric patients (0-1 year: 537; 1-4 years: 159; 5-11 years: 115; 12-18 years: 281).

# Missing data description

## Individual cases with missing data

| **Variable** | **Missing Count** | **Percentage of total cases** |
| --- | --- | --- |
| PHCC attendance | 0 | 0.00% |
| Age | 282 | 0.49% |
| Sex | 100 | 0.17% |
| Utstein location | 322 | 0.56% |
| Aetiology | 55 | 0.10% |
| Initial rhythm | 3,103 | 5.39% |
| Witness/bystander CPR status | 161 | 0.28% |
| Predicted survival | 3,528 | 6.12% |

Missing data heatmap. Blue indicates non-missing and pink indicates missing. Column of numbers on left of heat map indicates number of observations. Column of numbers on right indicates number of missing variables:

## Neighbourhood aggregated data missing data

| **Variable** | **Missing Count** | **Percentage of total cases** |
| --- | --- | --- |
| Total OHCA | 0 | 0.00% |
| OHCA incidence | 0 | 0.00% |
| Number of PHCC responses | 0 | 0.00% |
| Urban rural classification | 0 | 0.00% |
| Ambulance service | 0 | 0.00% |
| Predicted PHCC response time | 0 | 0.00% |
| Predicted HEMS team response time | 0 | 0.00% |
| High risk neighbourhood | 0 | 0.00% |
| IMD decile | 0 | 0.00% |
| Median predicted survival | 426 | 4.2% |
| Median observed PHCC response time | 3,301 | 32.9% |

Missing data heatmap. Blue indicates non-missing and pink indicates missing. Column of numbers on left of heat map indicates number of observations. Column of numbers on right indicates number of missing variables:

# Neighbourhood-level patient characteristics stratified by quartile of median predicted survival

| **Variable** | **Q1 – lowest predicted survival** | **Q2** | **Q3** | **Q4 – highest predicted survival** |
| --- | --- | --- | --- | --- |
| Number of neighbourhoods | 2,402 | 2,404 | 2,398 | 2,402 |
| Number of OHCA | 13,096 | 15,557 | 16,167 | 12,746 |
| Age – years (median) | 74 | 70 | 68 | 68 |
| Witness/bystander CPR (%)  Unwitnessed and no bystander CPR  Unwitnessed and bystander CPR provided  Bystander witnessed and bystander CPR provided  Bystander witnessed and no bystander CPR  EMS witnessed | 18.0  33.4  32.1  12.0  4.4 | 16.7  24.4  39.3  12.7  12.3 | 12.7  21.2  41.1  12.3  12.4 | 10.4  17.8  43.5  11.5  16.4 |
| Initial rhythm (%)  Shockable (VF/VT)  Asystole  PEA | 9.0  73.4  12.3 | 14.0  58.3  22.9 | 19.6  48.1  27.7 | 46.3  30.2  17.8 |
| Aetiology (%)  Medical  Asphyxia  Drowning  Overdose  Traumatic  Other (non-cardiac) | 93.7  1.6  2.9  0.4  2.9  1.0 | 93.8  2.1  0.3  0.7  2.2  0.8 | 91.8  2.6  0.4  1.8  2.2  1.1 | 93.0  1.8  0.3  2.1  1.8  0.9 |

Values are unweighted neighbourhood-level means for proportions and median for age. Quartiles are defined by neighbourhood median pre-EMS predicted survival (Q1 lowest to Q4 highest). Predicted survival was derived from age, aetiology, witnessed and bystander CPR status, and initial rhythm using a validated pre-EMS model.

# Observed prehospital critical care response

## Description of observed response

15,896 patients were attended by a PHCC asset. This encompassed 17,251 individual PHCC asset responses since some patients were attended by more than one PHCC asset. Of these responses, 7,337 (42.5%) were by helicopter. Physician-led HEMS assets attended 7,154 patients (45.0%), comprising 4,482 helicopter responses and 2,672 road responses, while paramedic-led HEMS assets attended 6,800 patients (42.8%), comprising 3,105 helicopter responses and 3,695 road responses. BASICS responders attended 2,126 OHCA incidents (13.4%), all by road. A single NHS physician‑led team attended 827 incidents (5.2%), all by road.

## Response times

### Histogram of observed prehospital critical care response times (from activation to on scene time)

Quartile 1 = 11.4 minutes, Median = 16.0 minutes, Quartile 3 = 21.6 minutes

### Histogram of observed helicopter response times (from activation to on scene time)

Quartile 1 = 13.6 minutes, Median = 17.9 minutes, Quartile 3 = 22.5 minutes

### Histogram of observed road response times (from activation to on scene time)

Quartile 1 = 10.0 minutes, Median = 14.4 minutes, Quartile 3 = 20.4 minutes

### Map of observed prehospital critical care response times (from activation to on scene time)

### Quintile map of observed prehospital critical care response times (from activation to on scene time)

# Base-specific predicted prehospital critical care response times

Firstly, to ensure predictions reflected local practices, all PHCC services supplied base-level operational parameters (usual helicopter model, median lift time, typical cruise speed). To capture which neighbourhoods close to a HEMS base would typically receive a road rather than helicopter response, a standard clinical case vignette was provided, describing a typical OHCA case where PHCC was dispatched and there were no restrictions on flying. Services reviewed a blinded grouped-value road response time map ([https://road-response-group-smooth.netlify.app/](https://eur01.safelinks.protection.outlook.com/?url=https%3A%2F%2Froad-response-group-smooth.netlify.app%2F&data=05%7C02%7CA.Boulton%40warwick.ac.uk%7Ca8273dc63d4c4255ee0f08ddbd4c9704%7C09bacfbd47ef446592653546f2eaf6bc%7C0%7C0%7C638874858833952031%7CUnknown%7CTWFpbGZsb3d8eyJFbXB0eU1hcGkiOnRydWUsIlYiOiIwLjAuMDAwMCIsIlAiOiJXaW4zMiIsIkFOIjoiTWFpbCIsIldUIjoyfQ%3D%3D%7C0%7C%7C%7C&sdata=NMgx%2BTTxexFCILxXXmLscnZPRYzg6wAkHOCbC5YVWd4%3D&reserved=0)) and indicated which response mode would be expected, based on local expertise of operational norms, local geography, and receiving hospitals. These preferences, along with the helicopter operational parameters, varied across services. Secondly, each neighbourhood was assigned its nearest PHCC base, along with the appropriate base-specific response mode (helicopter or road). Thirdly, helicopter response times were calculated using Euclidean distance, base-specific cruise speed and penalty for median lift time. These predictions were then calibrated using linear regression fitted on a subset of observed helicopter response times, adjusting for neighbourhood rural-urban classification. For road responses, Open Source Routing Machine (OSRM) was used to generate predicted travel times under standard road conditions. To account for emergency blue‑light driving, OSRM‑predicted travel times were compared with observed activation‑to‑on‑scene times for road‑attended cases. Percentage reductions from 0-50% (0.1% increments) were applied to predicted times, and the reduction yielding the lowest mean absolute error (7.7%) was selected. Overall predictive performance of individualised response times was evaluated with mean absolute error and root mean squared error measurements using cases in which the observed response mode matched expected response mode of the model (since the model assumed no restrictions on flying) and where there was no evidence that the responding asset originated from a base other than the nearest base as assumed in the model. Predictive performance is reported in the main manuscript and calibration graphs are reported below.

Below is a copy of the email sent to all PHCC services within the study region:

“

We are trying to predict response times for neighbourhoods. This is challenging and there are many moving parts.  With your help, we hope to develop a model that is both accurate and meaningful to services. One key aspect is identifying the area around each base where a road response would be faster than helicopter deployment. This will vary by base due to factors such as local road networks, aircraft type, and geography. Your local expertise is therefore essential.

Please consider the following scenario:

1. A 55-year-old male suffers a witnessed cardiac arrest after complaining of chest pain. There was immediate bystander CPR. The crew arrive 5 minutes later, and the initial rhythm is shockable. After 10 minutes of paramedic resuscitation, ROSC is achieved. The patient is now agitated, biting on an iGel, and is hypotensive. The crew requests critical care backup.
2. The helicopter is available and there are no restrictions on flying.

Please review the following map: [https://road-response-group-smooth.netlify.app/](https://eur01.safelinks.protection.outlook.com/?url=https%3A%2F%2Froad-response-group-smooth.netlify.app%2F&data=05%7C02%7Ca.boulton%40warwick.ac.uk%7C3f30a5640cd54e9ec1c308ddd58f3a8b%7C09bacfbd47ef446592653546f2eaf6bc%7C0%7C0%7C638901533363055475%7CUnknown%7CTWFpbGZsb3d8eyJFbXB0eU1hcGkiOnRydWUsIlYiOiIwLjAuMDAwMCIsIlAiOiJXaW4zMiIsIkFOIjoiTWFpbCIsIldUIjoyfQ%3D%3D%7C0%7C%7C%7C&sdata=1Zr3%2FuoyuNJtnSPa3nuwEXvzZC%2BOwUXDnLNX0BVwny0%3D&reserved=0)

**Within which group would you choose to respond to by road rather than helicopter?**

We would appreciate your response for each of your bases - please can you list.

Additionally, could you please provide the following information for each base:

1. The usual helicopter at each base (H135, H145, AW169 etc.)
2. The median lift time at each base
3. The typical cruise speed of the aircraft

“

# Predicted versus observed response times

# Histogram of predicted prehospital critical care response times

# Population distribution by prehospital critical care response time

# Population distribution by prehospital critical care response time across services

# Population distribution by HEMS-only configuration response time in service B

# Model performance across various model structures

Akaike information criterion (AIC). Lower AIC indicates better model fit. *Lowest AIC, and also the primary analysis.

|  | **High risk neighbourhood** | | **Pre-EMS predicted survival** | | **Index of multiple deprivation decile (ordinal)** | | **Index of multiple deprivation decile (continuous)** | |
| --- | --- | --- | --- | --- | --- | --- | --- | --- |
| **Model** | **Adjusted for rural-urban classification. AIC** | **Without adjustment for rural-urban classification. AIC** | **Adjusted for rural-urban classification. AIC** | **Without adjustment for rural-urban classification. AIC** | **Adjusted for rural-urban classification. AIC** | **Without adjustment for rural-urban classification. AIC** | **Adjusted for rural-urban classification. AIC** | **Without adjustment for rural-urban classification. AIC** |
| Nil service | 76848.7 | 77603.2 | 61705.9 | 61723.7 | 45772.6 | 46163.1 | 48827.1 | 49272.2 |
| Service random intercept | 76137.9 | 76776.8 | 61550.4 | 61568.2 | 45521.8 | 45889.8 | 48589.5 | 48997.5 |
| Service random intercept and slope | 75891.8 | 76507.5 | 61527.5 | 61548.2 | 45410.4 | 45776.2 | 48478.8 | 48872.2 |
| Service interaction | 75863.3* | 76478.8 | 61501.8* | 61524.9 | 45386.3* | 45735 | 48448.2 | 48845.3 |

Summary: Across all specifications, models including a service–response time interaction term consistently produced the lowest AIC values. Adjusting for rural-urban classification, also consistently produced the lowest AIC values. Modelling IMD as an ordinal variable also produced a consistently lower AIC value.

# Model performance across analyses

| **Model and variable** | **Akaike information criterion** | **Neighbourhoods in analysis** |
| --- | --- | --- |
| Primary analysis: Predicted critical care response and adjusted for rural-urban classification | | |
| High-risk neighbourhood | 75863.3 | 9639 |
| Pre-EMS predicted survival | 61501.8 | 9606 |
| Index of multiple deprivation decile | 45386.3 | 10032 |
| Secondary analysis: HEMS-only configuration and adjusted for rural-urban classification | | |
| High-risk neighbourhood | 75904.3 | 9639 |
| Pre-EMS predicted survival | 61507.5 | 9606 |
| Index of multiple deprivation decile | 45374 | 10032 |
| Sensitivity analysis: Median observed prehospital critical care response times, adjusted for rural-urban classification | | |
| High-risk neighbourhood | 60578.7 | 6731 |
| Pre-EMS predicted survival | 42621.2 | 6720 |
| Index of multiple deprivation decile | 30274 | 6731 |
| Sensitivity analysis: Road distance from nearest PHCC base, adjusted for rural-urban classification | | |
| High-risk neighbourhood | 75934 | 9639 |
| Pre-EMS predicted survival | 61522.3 | 9606 |
| Index of multiple deprivation decile | 45400.9 | 10032 |
| Sensitivity analysis: Straight-line distance from nearest HEMS base, adjusted for rural-urban classification | | |
| High-risk neighbourhood | 75948.1 | 9639 |
| Pre-EMS predicted survival | 61526.6 | 9606 |
| Index of multiple deprivation decile | 45352.6 | 10032 |

# Model diagnostic: Spatial autocorrelation in raw outcomes and model residuals

| **Analysis** | **Outcome** | **Moran’s I** |
| --- | --- | --- |
| Raw outcome | High-risk neighbourhood | 0.0608 |
| Raw outcome | Pre-EMS predicted survival | 0.0329 |
| Raw outcome | Neighbourhood deprivation (IMD) | 0.5406 |
| Primary analysis | High-risk neighbourhood | 0.0267 |
| Primary analysis | Pre-EMS predicted survival | 0.0118 |
| Primary analysis | Neighbourhood deprivation (IMD) | 0.4917 |
| Secondary analysis | High-risk neighbourhood | 0.0276 |
| Secondary analysis | Pre-EMS predicted survival | 0.0121 |
| Secondary analysis | Neighbourhood deprivation (IMD) | 0.4908 |

# Sensitivity analyses

## Sensitivity analysis: observed response time and distances

Green = favourable alignment; Amber = no significant relationship; Red = poor alignment.

| **Variable** | **Service A** | **Service B** | **Service C** |
| --- | --- | --- | --- |
| High risk neighbourhood (odds ratio, 95% confidence interval) | | | |
| Sensitivity analysis:  Observed PHCC response time (per 5 mins) (n=6731) | 1.073 (1.040 to 1.106) | 0.893 (0.869 to 0.917) | 0.979 (0.957 to 1.002) |
| Sensitivity analysis:  Road distance from nearest PHCC base (per 10 kilometres) (n=9639) | 1.123 (1.098 to 1.148) | 0.918 (0.901 to 0.936) | 1.054 (1.038 to 1.070) |
| Sensitivity analysis:  Straight-line distance from nearest HEMS base (per 10 kilometres) (n=9639) | 1.185 (1.151 to 1.220) | 1.089 (1.059 to 1.120) | 1.071 (1.050 to 1.092) |
| Pre-EMS predicted survival (coefficient, 95% confidence interval) | | | |
| Sensitivity analysis:  Observed PHCC response time (n=6720) | -0.110 (-0.322 to 0.102) | -0.008 (-0.195 to 0.179) | -0.138 (-0.290 to 0.014) |
| Sensitivity analysis:  Road distance from nearest PHCC base (per 10 kilometres) (n=9606) | -0.048 (-0.190 to 0.093) | 0.184 (0.061 to 0.307) | -0.187 (-0.285 to -0.089) |
| Sensitivity analysis:  Straight-line distance from nearest HEMS base (per 10 kilometres) (n=9609) | -0.075 (-0.258 to 0.108) | -0.044 (-0.230 to 0.141) | -0.270 (-0.395 to -0.144) |
| Neighbourhood deprivation (IMD) (odds ratio, 95% confidence interval) | | | |
| Sensitivity analysis:  Observed PHCC response time (n=6731) | 0.968 (0.908 to 1.032) | 1.227 (1.155 to 1.303) | 0.939 (0.897 to 0.984) |
| Sensitivity analysis:  Road distance from nearest PHCC base (per 10 kilometres) (n=10032) | 0.882 (0.847 to 0.919) | 1.198 (1.151 to 1.247) | 0.894 (0.867 to 0.922) |
| Sensitivity analysis:  Straight-line distance from nearest HEMS base (per 10 kilometres) (n=10032) | 0.813 (0.771 to 0.857) | 0.856 (0.823 to 0.890) | 0.743 (0.700 to 0.788) |

IMD: Index of multiple deprivation. For high‑risk neighbourhood, an OR >1 indicates higher chance of being a high‑risk neighbourhood per 5‑minute increase in response time. For neighbourhood pre-EMS predicted survival, coefficients represent the percentage‑point change in predicted survival per 5‑minute increase in response time. For IMD decile (1=most deprived, 10=least deprived), an OR >1 indicates the neighbourhood being less deprived per 5-minute increase in response time; OR <1 indicates the neighbourhood being more deprived per 5-minute increase in response time.

## Sensitivity analysis: excluding cases from COVID-19 pandemic peaks

Peak one of the COVID pandemic was considered to be 16^th^ March 2020 until 30^th^ April 2020 and peak two was considered to be 15^th^ December 2020 to 15^th^ February 2021. Cases occurring within these periods (inclusive) were excluded from the following sensitivity analysis. There were 54,355 cases across 9,611 neighbourhoods.

Green = favourable alignment; Amber = no significant relationship; Red = poor alignment.

| **Variable** | **Service A** | **Service B** | **Service C** |
| --- | --- | --- | --- |
| High risk neighbourhood (odds ratio, 95% confidence interval) | | | |
| Sensitivity analysis:  Excluding COVID-19 pandemic peaks | 1.489  (1.404 to 1.579) | 0.841  (0.805 to 0.878) | 1.164  (1.119 to 1.210) |
| Pre-EMS predicted survival (coefficient, 95% confidence interval) | | | |
| Sensitivity analysis:  Excluding COVID-19 pandemic peaks | -0.186  (-0.556 to 0.184) | 0.362  (0.074 to 0.651) | -0.766  (-1.015 to -0.518) |

For high‑risk neighbourhood, an OR >1 indicates higher chance of being a high‑risk neighbourhood per 5‑minute increase in response time. For neighbourhood pre-EMS predicted survival, coefficients represent the percentage‑point change in predicted survival per 5‑minute increase in response time.

## Sensitivity analysis: with adjustment for EMS response time

Green = favourable alignment; Amber = no significant relationship; Red = poor alignment. Individualised predicted PHCC response times are assigned from each neighbourhood's nearest PHCC base across three ambulance service regions, with response mode (road or helicopter) determined by base-specific operational norms reflecting local practice and geography. Predictions incorporate base-level helicopter parameters (lift time, cruise speed) and are calibrated against observed times using linear regression with a rural-urban adjustment. The HEMS-only configuration removes the urban NHS car asset from Service B.

| **Variable** | **Service A** | **Service B** | **Service C** |
| --- | --- | --- | --- |
| High risk neighbourhood (odds ratio, 95% confidence interval) | | | |
| Primary analysis:  Predicted PHCC response time (n=9639) | 1.576  (1.486 to 1.672) | 0.893  (0.854 to 0.933) | 1.167  (1.122 to 1.214) |
| Secondary analysis:  Predicted HEMS-only response time (n=9639) | 1.585  (1.494 to 1.681) | 1.192  (1.124 to 1.216) | 1.169  (1.124 to 1.216) |
| Pre-EMS predicted survival (coefficient, 95% confidence interval) | | | |
| Primary analysis:  Predicted PHCC response time (n=9606) | -0.094  (-0.463 to 0.275) | 0.473  (0.184 to 0.761) | -0.763  (-1.010 to -0.515) |
| Secondary analysis:  Predicted HEMS-only response time (n=9606) | -0.104  (-0.474 to 0.265) | -0.047  (-0.438 to 0.344) | -0.767  (-1.015 to -0.519) |
| Neighbourhood deprivation (IMD) (odds ratio, 95% confidence interval) | | | |
| Primary analysis:  Predicted PHCC response time (n=9639) | 0.639  (0.572 to 0.714) | 1.228  (1.116 to 1.352) | 0.687  (0.634 to 0.744) |
| Secondary analysis:  Predicted HEMS-only response time (n=9639) | 0.632  (0.566 to 0.706) | 0.658  (0.581 to 0.745) | 0.683  (0.631 to 0.740) |

Effect estimates are per 5-minute increase in response time. IMD: Index of multiple deprivation. For high‑risk neighbourhood, an OR >1 indicates higher chance of being a high‑risk neighbourhood per 5‑minute increase in response time. For neighbourhood pre-EMS predicted survival, coefficients represent the percentage‑point change in predicted survival per 5‑minute increase in response time. For IMD decile (1=most deprived, 10=least deprived), an OR >1 indicates the neighbourhood being less deprived per 5-minute increase in response time; OR <1 indicates the neighbourhood being more deprived per 5-minute increase in response time.

## Sensitivity analysis: without adjustment for rural-urban classification

Green = favourable alignment; Amber = no significant relationship; Red = poor alignment. Individualised predicted PHCC response times are assigned from each neighbourhood's nearest PHCC base across three ambulance service regions, with response mode (road or helicopter) determined by base-specific operational norms reflecting local practice and geography. Predictions incorporate base-level helicopter parameters (lift time, cruise speed) and are calibrated against observed times using linear regression with a rural-urban adjustment. The HEMS-only configuration removes the urban NHS car asset from Service B.

| **Variable** | **Service A** | **Service B** | **Service C** |
| --- | --- | --- | --- |
| High risk neighbourhood (odds ratio, 95% confidence interval) | | | |
| Primary analysis:  Predicted PHCC response time (n=9639) | 1.394 (1.316 to 1.477) | 0.781 (0.748 to 0.815) | 1.122 (1.079 to 1.166) |
| Secondary analysis:  Predicted HEMS-only response time (n=9639) | 1.394 (1.316 to 1.477) | 1.060 (1.000 to 1.124) | 1.122 (1.079 to 1.166) |
| Pre-EMS predicted survival (coefficient, 95% confidence interval) | | | |
| Primary analysis:  Predicted PHCC response time (n=9606) | -0.266 (-0.635 to 0.102) | 0.263 (-0.023 to 0.549) | -0.811 (-1.059 to -0.563) |
| Secondary analysis:  Predicted HEMS-only response time (n=9606) | -0.266 (-0.635 to 0.102) | -0.199 (-0.590 to 0.192) | -0.811 (-1.059 to -0.563) |
| Neighbourhood deprivation (IMD) (odds ratio, 95% confidence interval) | | | |
| Primary analysis:  Predicted PHCC response time (n=10032) | 0.740 (0.664 to 0.824) | 1.453 (1.325 to 1.594) | 0.729 (0.675 to 0.787) |
| Secondary analysis:  Predicted HEMS-only response time (n=10032) | 0.741 (0.665 to 0.825) | 0.785 (0.695 to 0.887) | 0.729 (0.676 to 0.787) |

Effect estimates are per 5-minute increase in response time. IMD: Index of multiple deprivation. For high‑risk neighbourhood, an OR >1 indicates higher chance of being a high‑risk neighbourhood per 5‑minute increase in response time. For neighbourhood pre-EMS predicted survival, coefficients represent the percentage‑point change in predicted survival per 5‑minute increase in response time. For IMD decile (1=most deprived, 10=least deprived), an OR >1 indicates the neighbourhood being less deprived per 5-minute increase in response time; OR <1 indicates the neighbourhood being more deprived per 5-minute increase in response time.

## Sensitivity analysis: Modelling of Index of Multiple Deprivation as a continuous variable

Green = favourable alignment; Amber = no significant relationship; Red = poor alignment. Modelling of Index of Multiple Deprivation as a continuous variable. Index of multiple deprivation decile (1=most deprived, 10=least deprived), therefore coefficient >0 indicates the neighbourhood being less deprived per 5-minute increase in response time; coefficient <0 indicates the neighbourhood being more deprived per 5-minute increase in response time.

| **Response time variable** | **Adjustment for rural-urban classification** | **Service A** | **Service B** | **Service C** | **Neighbourhoods in analysis** |
| --- | --- | --- | --- | --- | --- |
| Primary analysis: All prehospital critical care | Unadjusted | -0.447 (-0.613 to -0.281) | 0.595 (0.444 to 0.747) | -0.506 (-0.630 to -0.383) | 10032 |
| Primary analysis: All prehospital critical care | Adjusted | -0.601 (-0.764 to -0.437) | 0.399 (0.249 to 0.549) | -0.607 (-0.728 to -0.485) | 10032 |
| Secondary analysis: HEMS services only | Unadjusted | -0.447 (-0.614 to -0.281) | -0.447 (-0.644 to -0.249) | -0.506 (-0.630 to -0.383) | 10032 |
| Secondary analysis: HEMS services only | Adjusted | -0.610 (-0.774 to -0.447) | -0.633 (-0.827 to -0.440) | -0.613 (-0.734 to -0.491) | 10032 |

## Sensitivity analysis: Spatial error model of Index of Multiple Deprivation as a continuous variable

Green = favourable alignment; Amber = no significant relationship; Red = poor alignment. Spatial error model due to spatial autocorrelation in deprivation. The spatial error model was fitted using continuous IMD given the constraints of available spatial regression implementations for ordinal outcomes. Index of multiple deprivation decile (1=most deprived, 10=least deprived), therefore coefficient >0 indicates the neighbourhood being less deprived per 5-minute increase in response time; coefficient <0 indicates the neighbourhood being more deprived per 5-minute increase in response time.

| **Response time variable** | **Service A** | **Service B** | **Service C** | **Residuals Moran’s I** |
| --- | --- | --- | --- | --- |
| Primary analysis: All prehospital critical care | -0.393 (-0.798 to 0.012) | 0.300 (-0.067 to 0.668) | -0.438 (-0.708 to -0.168) | -0.0355 (p>0.05) |
| Secondary analysis: HEMS services only | -0.411 (-0.817 to -0.006) | -0.692 (-1.176 to -0.207) | -0.447 (-0.717 to -0.177) | -0.0354 (p>0.05) |
